# Pro-Inflammatory Effects of Indoxyl Sulfate in Mice: Impairment of Intestinal Homeostasis and Immune Response

**DOI:** 10.3390/ijms22031135

**Published:** 2021-01-24

**Authors:** Shara Francesca Rapa, Francesco Prisco, Ada Popolo, Valentina Iovane, Giuseppina Autore, Biagio Raffaele Di Iorio, Fabrizio Dal Piaz, Orlando Paciello, Fuyu Nishijima, Stefania Marzocco

**Affiliations:** 1 Department of Pharmacy, University of Salerno, 84084 Fisciano, SA, Italy; srapa@unisa.it (S.F.R.); apopolo@unisa.it (A.P.); viovane@unisa.it (V.I.); autore@unisa.it (G.A.); 2 Department of Veterinary Medicine and Animal Production, University of Naples Federico II, 80137 Napoli, NA, Italy; francesco.prisco@unina.it (F.P.); paciello@unina.it (O.P.); 3 UOC Nephrology AORN “San Giuseppe Moscati”, C.da Amoretta, 83100 Avellino, AV, Italy; br.diiorio@gmail.com; 4 Department of Medicine and Surgery, University of Salerno, 84084 Fisciano, SA, Italy; fdalpiaz@unisa.it; 5 Pharmaceuticals Division, Kureha Corporation, Tokyo 169-8503, Japan; fy.nishijima1955@gmail.com

**Keywords:** indoxyl sulfate, chronic kidney disease, intestinal inflammation, oxidative stress, intestinal epithelial cells, primary murine peritoneal macrophages

## Abstract

The intestines are recognized as the main source of chronic inflammation in chronic kidney disease (CKD) and, among other cells, macrophages are involved in modulating this process as well as in the impaired immune response which also occurs in CKD patients. In this study, we evaluated the effect of Indoxyl Sulfate (IS), a protein bound uremic toxin poorly eliminated by hemodialysis, on inflammatory, oxidative stress and pro-apoptotic parameters, at the intestinal level in mice, on intestinal epithelial cells (IEC-6) and on primary murine peritoneal macrophages. C57BL/6J mice were treated with IS (800 mg/kg i.p.) for 3 or 6 h and histopathological analysis showed that IS induced intestinal inflammation and increased cyclooxygenase-2 (COX-2), nitrotyrosine and Bax expression in intestinal tissue. In IEC-6 cells, IS (125–1000 µM) increased tumor necrosis factor-α levels, COX-2 and inducible nitric oxide synthase expression and nitrotyrosine formation. Moreover, IS increased pro-oxidant, pro-inflammatory and pro-apoptotic parameters in peritoneal macrophages from IS-treated mice. Also, the serum concentration of IS and pro-inflammatory levels of cytokines resulted increased in IS-treated mice. Our results indicate that IS significantly contributes to affect intestinal homeostasis, immune response, and to induce a systemic pro-inflammatory state thus highlighting its potential role as therapeutic target in CKD patients.

## 1. Introduction

Chronic kidney disease (CKD) is associated with a persistent systemic inflammation and acquired immunodeficiency which promote the pathogenesis of many CKD-associated complications as leading causes of death [1,2,3]. With respect to chronic inflammation, a multitude of dialysis- and non-dialysis-related factors, including infection, intravenous iron administration, blood dialyzer interface and preexisting heart failure, can play a role [4,5]. It is interesting to note that, despite technical innovations over recent decades (e.g., biocompatible dialysis membranes, no toxic equipment sterilization, etc.), the systemic inflammation, with its resulting oxidative stress, has persisted in CKD patients [6]. The previous view that the intestine is a largely inert organ has been deeply changed and accumulating evidence highlighted that a chronic inflammatory state is a nontraditional risk factor in CKD patients and indicated that the gastrointestinal tract plays a pivotal role in systemic inflammation occurring in these patients [7,8]. 

Gut bacterial DNA fragments have been detected in the blood of both pre-dialysis CKD and chronic hemodialysis patients [9]. Endotoxin, derived from the cell wall of Gram-negative bacteria, is measurable in the blood of dialysis patients and correlates with the severity of systemic inflammation in the absence of clinically detectable infection [10]. Indeed, levels of circulating endotoxin increase with the severity of CKD stage and are most elevated in chronic hemodialysis and peritoneal dialysis patients [11,12]. The intestinal epithelium has an important role by forming a physical and biochemical barrier to commensal and pathogenic microorganism. In fact, an impaired intestinal epithelial barrier can induce a translocation of gut bacteria and bacterial components into the circulation, which can, in turn, activate innate immunity and systemic inflammation [9,13]. A pivotal role in CKD-associated complications is attributed to the progressive retention of a large number of compounds which, under normal conditions, are excreted by healthy kidneys. These metabolites, called “uremic toxins”, have harmful effects in various physiological functions in CKD patients including immune response impairment [14,15,16,17,18,19] and intestinal homeostasis alterations. The accumulation of uremic toxins, such as indoxyl sulfate (IS), is implicated in the progression of renal failure and in its associated complications. Data indicate that serum IS levels are markedly increased in patients with renal disease and are related to disease severity. A trial involving patients at various stages of CKD found that IS levels were inversely related to renal function and directly related to aortic calcification and pulse wave velocity. Furthermore, elevated IS levels were associated with increased mortality in these patients [20]. IS is a uremic toxin resulting from the metabolism of dietary tryptophan. Tryptophan is metabolized into indole by intestinal bacteria and after intestinal absorption, indole is further converted into IS in the liver [8,21]. IS is a protein bound uremic toxin, due to its high-affinity binding to albumins, and, therefore, is poorly eliminated by dialysis treatment. IS values in CKD patients can increase even 50-fold compared to healthy people. IS has been considered a nephro-vascular toxin that causes nephrotoxicity especially on tubular cells, inhibits proliferation of endothelial cells [21,22,23] but it is involved also in other CKD-associated complications such as neurodegeneration [24,25]. 

Considering the involvement of IS in various CKD-associated complications a growing interest is pointed towards this gut-derived uremic toxin. Our group studied the effect of IS in vitro cellular models on intestinal epithelial barrier and on macrophages [26,27]. Here, we investigated the effect of IS in vivo, focusing the outcome of this uremic toxin on intestinal inflammatory and oxidative stress response, on intestinal epithelial cells (IECs) and evaluating its effects on peritoneal macrophage response. 

## 2. Results

### 2.1. IS Serum Levels 

IS (800 mg/kg) administration resulted in a significant increase in IS serum level giving a concentration of 85.15 ± 10.2 µM after 3 h (*p* < 0.001 vs. SHAM group—value 9.61 ± 0.38 µM) and of 50.74 ± 7.27 µM after 6 h (*p* < 0.01 vs. SHAM group). Moreover, IS serum levels after 6 h from the uremic toxin administration was significantly lower respect to the IS level observed after 3 h (*p* < 0.05). 

### 2.2. Histopathological Examination 

**Figure 1:**
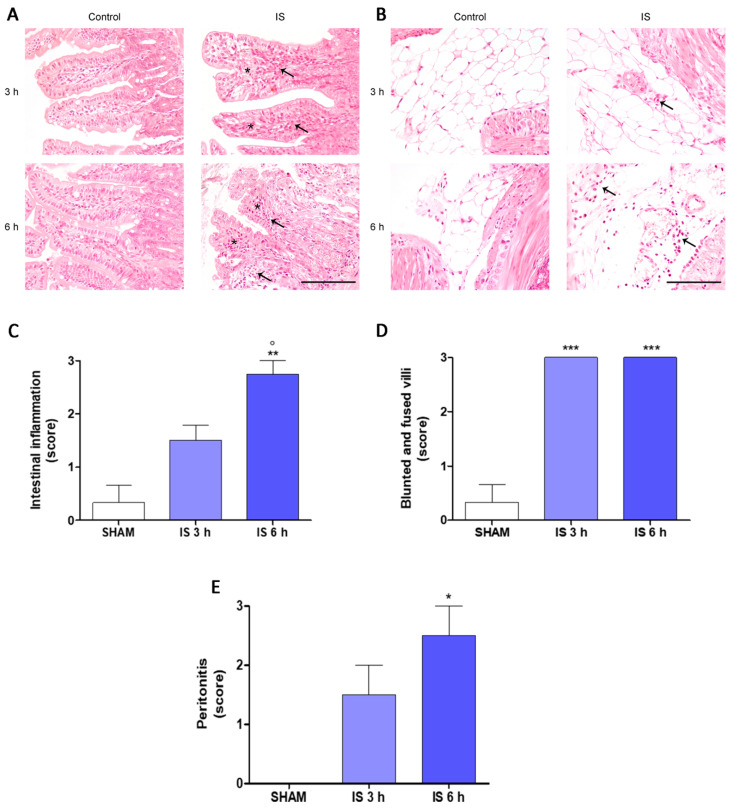
Histological examination of intestine (**A**) and mesenteries (**B**) of Indoxyl Sulfate (IS; 800 mg/kg; i.p.)-treated mice, after 3 h and 6 h from treatment (Hematoxylin and Eosin, 40× magnification, Scale Bar = 100 µm). (**A**) Mice treated with IS showed blunted and fused villi (asterisks) and infiltration of mononuclear inflammatory cells in the lamina propria (arrow) both 3 and 6 h after treatment. (**B**) The mesenteries of mice treated with IS were infiltrated by inflammatory cells (arrow) both 3 and 6 h after treatment. Score of intestinal inflammation (**C**), blunted and fused villi (**D**), and peritonitis (**E**). ***, ** and * denote respectively *p* < 0.001, *p* < 0.01 and *p* < 0.05 vs. SHAM; ° denotes *p* < 0.05 vs. IS 3 h.

In this study, we performed in vivo experiments, through the intraperitoneal administration of IS (800 mg/kg) in mice and the evaluation of various inflammatory parameters in two experimental times: after 3 h and 6 h from the administration of the uremic toxin. To characterize the inflammatory changes related to IS administration, a histopathological examination of the small intestine was performed as shown in Figure 1. In the treated animals, the examined intestines showed a mild to severe infiltration of mononuclear inflammatory cells mainly composed of lymphocytes and mainly located in the lamina propria of the villi. Blunted and fused villi and mild intestinal lymphangiectasia were also evident (Figure 1A). Moreover, the mesenteries of mice treated with IS were infiltrated by mononuclear inflammatory cells (peritonitis; Figure 1B). These alterations were occasionally observed or were absent in control mice.

The intestinal inflammation was more severe after 6 h of IS administration compared with 3 h administration of IS (*p* < 0.05 vs. IS 3 h) and compared with controls (*p* < 0.01 vs. SHAM; Figure 1C). Blunted and fused villi were more frequent in IS mice compared with controls both at 3 h (*p* < 0.001 vs. SHAM) and 6 h (*p* < 0.001 vs. SHAM) after the treatments (Figure 1D). Peritonitis was more severe in IS mice 6 h after treatment compared with IS mice 3 h after administration (*p* < 0.05 vs. IS 3 h; Figure 1E).

### 2.3. Immunohistochemical Localization of COX-2, Nitrotyrosine and BAX

**Figure 2:**
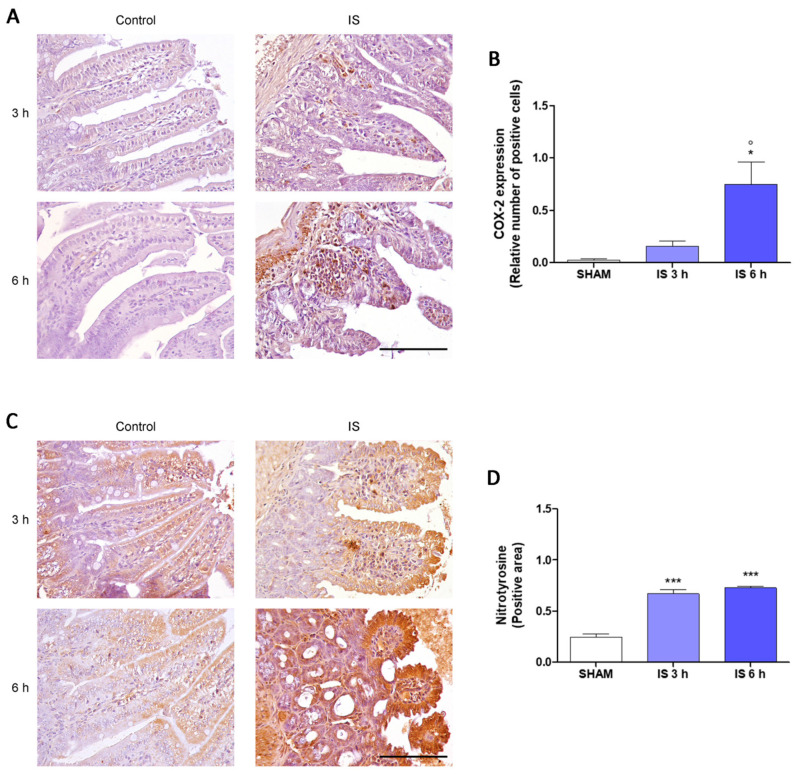
Immunohistochemical localization of COX-2 (**A**,**B**) and nitrotyrosine (**C**,**D**) of Indoxyl Sulfate (IS; 800 mg/kg; i.p.)-treated mice, after 3 h and 6 h of treatment (40× magnification, Scale Bar = 100 µm). *** and * denote respectively *p* < 0.001, and *p* < 0.05 vs. SHAM; ° denote *p* < 0.05 vs. IS 3 h.

**Figure 3:**
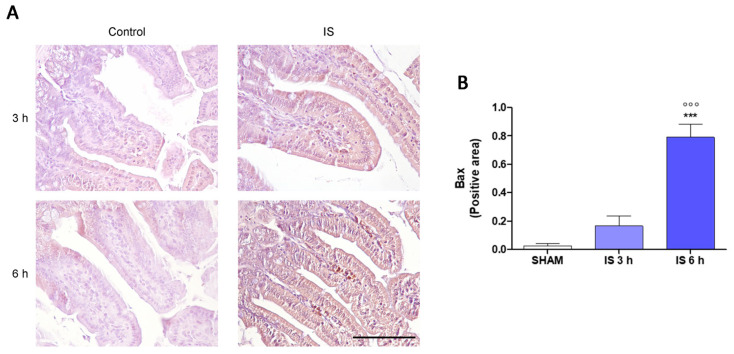
Immunohistochemical localization of Bax (**A**,**B**) of Indoxyl Sulfate (IS; 800 mg/kg; i.p.)-treated mice, after 3 h and 6 h of treatment (40× magnification, Scale Bar = 100 µm). *** denote *p* < 0.001 vs. SHAM; °°° denote *p* < 0.001 vs. IS 3 h.

To further explore the mechanism underlying intestinal changes in IS-treated mice, we assessed the immunohistochemical expression of COX-2, nitrotyrosine, and Bax. In the axis of the villi of the treated mice a greater number of COX-2-positive infiltrating inflammatory cells were observed compared to the SHAM group, in particular after 6 h from the treatment (*p* < 0.05 vs. SHAM and IS 3 h; Figure 2A,B). Inflammatory cells infiltrating the axis of the villi and epithelial cells of the intestinal mucosa showed a high immunohistochemical positivity to the anti-nitrotyrosine antibody in the animals treated with IS compared to the controls both 3 h and 6 h after treatment (*p* < 0.001 vs. SHAM; Figure 2C,D). The BAX expression of the inflammatory cells infiltrating the axis of the villi and of the epithelial cells of the intestinal mucosa was more intense in mice 6 h after IS treatment compared with SHAM and IS 3 h (*p* < 0.001; Figure 3A,B).

### 2.4. IS Enhanced Pro-Inflammatory Parameters, Especially in Inflammatory Conditions

**Figure 4:**
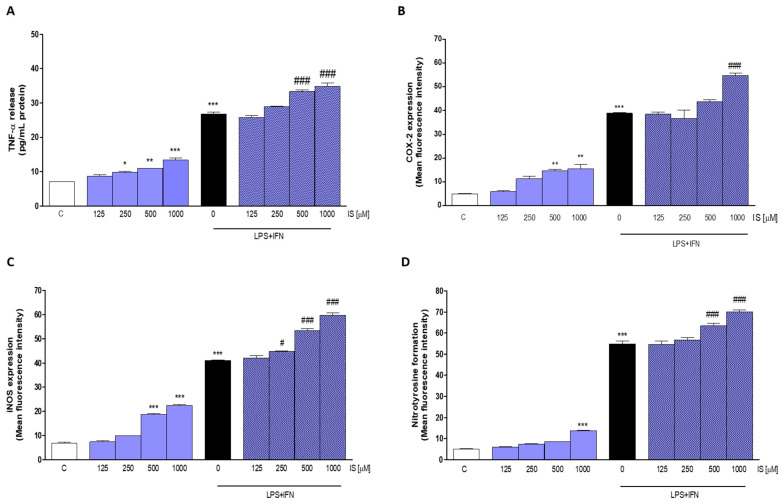
Effect of Indoxyl Sulfate (IS; 125–1000 µM) on pro-inflammatory mediators in lipopolysaccharide (LPS) + interferon-γ (IFN)-treated IEC-6 cells. TNF-α release (**A**), evaluated by ELISA assay, COX-2 (**B**) and iNOS (**C**) expression and nitrotyrosine formation (**D**), evaluated by the cytofluorimetric technique. Data are expressed as pg/mL or mean of fluorescence intensity. C denotes control group. ***, ** and * denote respectively *p* < 0.001, *p* < 0.01 and *p* < 0.05 vs. C; ### and # denote respectively *p* < 0.001 and *p* < 0.05 vs. LPS + IFN.

In order to further investigate the pro-inflammatory activity of IS at intestinal level, we performed in vitro experiments on IECs using the non-tumorigenic cell line IEC-6. IEC-6 cells were treated with IS (125–1000 µM), both alone and in the presence of inflammatory stimuli, such as LPS + IFN, with the aim to evaluate whether the IS could exacerbate an inflammatory state already in progress. The obtained results showed that IS significantly increased TNF-α levels in IEC-6 (*p* < 0.05 vs. C; Figure 4A), and interestingly, this release was further enhanced in inflammatory conditions (500 and 1000 µM *p* < 0.001 vs. LPS + IFN; Figure 4A). 

Moreover, IS significantly increased, at the highest concentrations tested, the expression of two enzymes primarily involved in inflammatory reactions at intestinal level, such as COX-2 and iNOS. Our data indicated a significant increase in COX-2 expression (*p* < 0.01 vs. C; Figure 4B), even in the presence of LPS + IFN (*p* < 0.001 vs. LPS + IFN), and in iNOS expression (*p* < 0.001 vs. C; Figure 4C). Also, in this case, the expression of the iNOS enzyme was increased in inflammatory conditions (*p* < 0.05 vs. LPS + IFN). 

Nitrotyrosine, a product of tyrosine nitration mediated by reactive nitrogen species such as peroxynitrite anion and nitrogen dioxide, is well-known marker of nitric oxide-dependent oxidative stress. In our study we evaluated the influence of IS on nitrotyrosine formation in IEC-6 cells, also in presence of LPS + IFN. A significant increase in nitrotyrosine formation was observed in IS-treated IEC-6 both in normal and in inflammatory conditions (*p* < 0.001 vs. LPS + IFN; Figure 4D).

### 2.5. IS Increased Pro-Inflammatory, Pro-Oxidant and Pro-Apoptotic Parameters in Primary Murine Peritoneal Macrophages 

**Figure 5:**
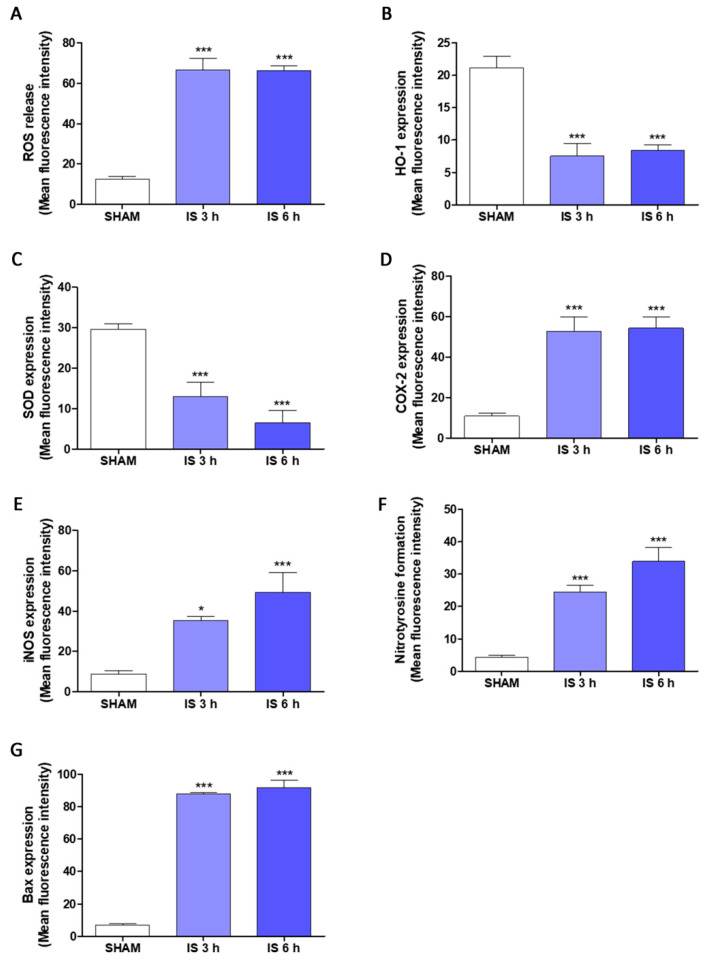
Effect of Indoxyl Sulfate (IS; 800 mg/kg; i.p.) on intracellular ROS release (**A**), evaluated by H_2_DCF-DA assay, HO-1 (**B**), SOD-2 (**C**), COX-2 (**D**), iNOS (**E**), nitrotyrosine (**F**), and Bax (**G**), evaluated by the cytofluorimetric technique, on primary murine peritoneal macrophages. Data are expressed as mean of fluorescence intensity. *** and * denote respectively *p* < 0.001 and *p* < 0.05 vs. SHAM.

After investigating the pro-inflammatory potential of IS at intestinal level, both in vivo and on IEC-6 cells, we evaluated the ability of IS in modulating inflammatory, oxidative and apoptotic parameters in primary macrophages deriving from the peritoneum of IS-treated mice, after 3 h and 6 h from the uremic toxin administration. Our results highlighted a significant increase in ROS release, index of oxidative stress, both after 3 h and 6 h-IS treatment in mice peritoneal macrophages (*p* < 0.001 vs. SHAM; Figure 5A). The expression of two enzymes with cytoprotective and antioxidant activity, such as HO-1 and SOD-2, was also evaluated. IS has been shown to have pro-oxidant activity, due to the inhibition of HO-1 (*p* < 0.001 vs. SHAM; Figure 5B) and SOD-2 (*p* < 0.001 vs. SHAM; Figure 5C). 

Cytofluorimetric analysis showed that IS treatment was also related to a significant increase of the two pro-inflammatory enzymes COX-2 and iNOS, at both experimental times (*p* < 0.05 vs. SHAM; Figure 5D,E). Furthermore, the formation of nitrotyrosine was also positively modulated by IS in primary peritoneal macrophages (*p* < 0.001 vs. SHAM; Figure 5F). 

A pro-apoptotic marker, the Bax protein, was also analyzed by flow cytometric techniques. IS demonstrated to significantly increase Bax expression both after 3 h and after 6 h (*p* < 0.001 vs. SHAM; Figure 5G).

### 2.6. Cytokines Levels in Mice Serum

**Figure 6:**
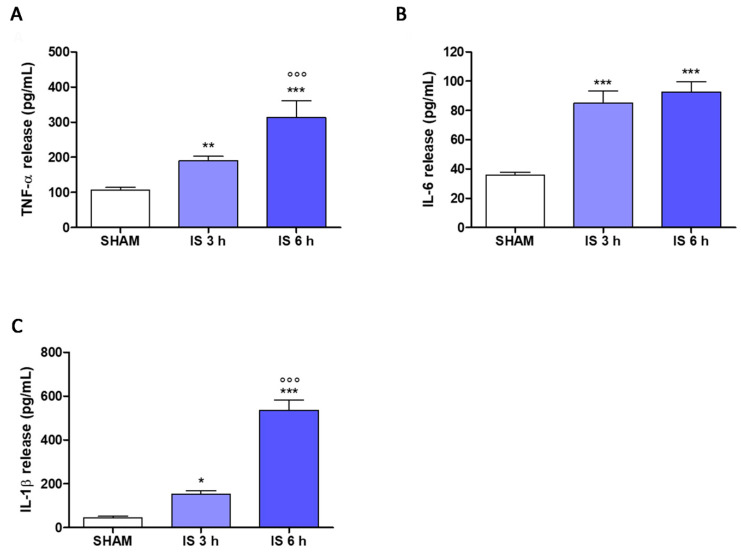
Effect of Indoxyl Sulfate (IS; 800 mg/kg; i.p.) on tumor necrosis factor-α (TNF-α; (**A**)), interleukin-6 (IL-6; (**B**)) and interleukin-1β (IL-1β; (**C**)) levels in mice serum after 3 h or 6 h of treatment, evaluated by Enzyme-Linked Immuno Sorbent Assay (ELISA). Values are expressed as pg/mL of cytokines release. ***, ** and * denote respectively *p* < 0.001, *p* < 0.01 and *p* < 0.05 vs. SHAM; °°° denotes *p* < 0.001 vs. IS 3 h.

It was interesting to evaluate the pro-inflammatory cytokines serum levels, such as TNF-α, IL-6, and IL-1β in our study. The results indicated that IS induced a significant increase in TNF-α levels (*p* < 0.01 vs. SHAM; *p* < 0.001 vs. IS 3 h; Figure 6A), but also a significant increase in IL-6 (*p* < 0.001 vs. SHAM; Figure 6B) and IL-1β (*p* < 0.05 vs. SHAM; *p* < 0.001 vs. IS 3 h; Figure 6C) serum levels both following treatment with IS at 3 h, and at 6 h. Interestingly, we observed a significant increase in TNF-α and IL-1β at 6 h vs. 3 h-IS treatment indicating a significant time related effect on these pro-inflammatory cytokines at the systemic level.

## 3. Discussion

Although substantial improvements have been made in clinical care, CKD remains a major public health burden, affecting 10–15% of the population, and its prevalence is constantly growing [28]. Persistent, low-grade inflammation is considered a CKD hallmark feature, being involved in the development of all-cause mortality of these patients especially related to cardiovascular disease and infections [29,30]. In order to reduce the inflammatory state different approaches have been proposed [31,32]; despite this, many factors contribute to the setting of the inflammatory status in CKD, including increased production of pro-inflammatory cytokines, oxidative stress and acidosis, chronic and recurrent infections, altered metabolism of adipose tissue, and finally, gut impaired homeostasis [30,33]. 

In this study we report that IS administration in mice induces intestinal inflammation and significantly contribute to a systemic inflammatory state by: (i) impairing intestinal homeostasis, (ii) activating a pro-inflammatory response in IECs, and (iii) inducing in peritoneal macrophages a pro-inflammatory and pro-oxidant response. 

In according with previous studies [24,34] our data indicated that IS i.p. administration resulted in a significant IS serum accumulation, observed mostly after 3 h from i.p. injection that decreases after 6 h. The observed decrease after 6 h is due to the normal renal function in mice thus, IS is rapidly excreted by glomerular filtration and tubular secretion [34]. 

Vaziri and colleagues reported a chronic inflammation state throughout the gastrointestinal tract—extending from esophagus to large bowel—in CKD patients in autopsy studies [35]. In our experiments, the histopathological analysis indicated that IS administration induced intestinal inflammation. In IS-treated animal, both after 3 h and 6 h, the examined intestinal tissue showed a mild to severe infiltration of the lamina propria by mononuclear inflammatory cells mainly composed of lymphocytes. Moreover, blunted and fused villi were more frequent in treated animals compared with controls, further suggesting an IS-induced mucosal damage [36]. 

Histopathological changes of the intestine were consistent with previous observation even if described in animal model of CKD induced by nephrectomy [34,37,38]. Furthermore, as we observed, evidence indicates that in CKD the inflammatory state of the gastrointestinal tract was associated with oxidative stress. The mechanisms underlying this process are still not clear, however, different animal models of CKD suggest that the IS-induced increased intestinal permeability allows the translocation of bacterial-derived toxins and antigenic molecules, such as LPS and bacterial DNA into the lamina propria of the intestine and subsequently into the systemic circulation [26,39]. These molecules have chemotactic activity for inflammatory cells, possibly explaining the intestinal inflammation [39].

A mild peritonitis was seen in IS mice both 3 h and 6 h after treatment but not statistically differences were seen compared with controls. These findings are consistent with the peritoneal inflammation reported both in rat model of uremia [40] and in humans before starting peritoneal dialysis treatment [41]. The cause of the peritoneal inflammatory infiltrate is still elusive. However, the translocation of bacterial antigenic molecules from the intestinal lumen, due to the increased intestinal epithelial permeability, may be implied [41].

Among pro-inflammatory enzymes, COX-2 is involved in many intestinal diseases [42,43] and it is associated to an immediate-early response, being normally absent from most cells but induced in response to inflammatory stimuli mainly at sites of inflammation. Our data indicated that COX-2 expression was not evident in intestinal epithelial cells, however, a higher number of COX-2-positive infiltrating inflammatory cells were observed in the axis of the villi of the treated mice, mostly after 6 h of treatment. These results are in agreement with previous studies, which reported an upregulation of pro-inflammatory parameters, such as the COX-2 enzyme at intestinal level, during CKD [37]. In CKD-associated intestinal inflammation an increase in iNOS expression was also reported [44] and intestinal inflammation is also characterized by an increased nitric oxide (NO) release and nitrotyrosine formation, a product of tyrosine nitration mediated by reactive nitrogen species such as peroxynitrite anion and nitrogen dioxide, is considered a marker of NO-dependent oxidative stress. Our results indicated that nitrotyrosine is expressed by inflammatory cells infiltrating the axis of the villi and that its expression is higher in cells of the epithelium of the intestinal mucosa in IS-treated mice compared with controls. The resulting intestinal inflammation is also associated to an increase in IECs apoptosis, mainly driven by pro-inflammatory mediators, that compromises barrier integrity, leading to a further inflammatory condition [45,46]. Our data indicated that the IS-resulting damage of the IECs was also associated to an increase in the pro-apoptotic protein Bax expression, mostly after 6 h of treatment, thus further supporting the IS-effect in impairing intestinal homeostasis even when its serum concentration decreases. 

The intestinal epithelium serves as an immunological guard forming a barrier between the intestinal lumen, containing microbiota and their derivatives and metabolites, and the internal milieu [9,13]. In this scenario IECs play a pivotal role thus, their role let us to specifically investigate the effect of IS using IEC-6 cells as experimental cellular model. 

TNF-α is one of the major cytokines playing a pivotal role in intestinal inflammation and, in this condition, it is also significantly upregulated in IECs [47]. Similarly, the levels of pro-inflammatory enzymes such as COX-2 and iNOS are also increased in IECs, as a result of the intestinal inflammation, and contribute to the amplification of the inflammatory response (also via TNF-α) and to the oxidative and nitrosative stress [48,49]. 

In IEC-6 cells IS treatment induced a significant increase in TNF-α release, COX-2 and iNOS expression as well as in nitrotyrosine formation, thus indicating IECs as a target of IS-induced intestinal inflammation. Interestingly, these factors were further enhanced in IS treated IEC-6 cells in presence of LPS + INF, especially for TNF-α, suggesting a possible further damage induced by IS on IECs in presence of infections, a condition often occurring in CKD patients [50].

Intestinal inflammation can be further promoted by macrophages, also through pro-inflammatory cytokine production [51], thus, we also studied the status of peritoneal macrophages from IS-treated mice. Our results indicated that peritoneal macrophages from IS-treated mice were characterized by an oxidative stress state due to a significant increase in ROS release associated to an inhibition of the cytoprotective enzymes HO-1 and SOD-2 expression both after 3 h and 6 h. These data are in according with previous studies reporting the effect of IS in reducing these antioxidant enzymes, as well their related transcription factor, nuclear factor (erythroid-derived 2)-like 2 (Nrf2), in different organs and tissue [24,26,52]. Nrf-2 activation and the expression of its related cytoprotective enzymes are severely impaired in CKD and their activation could have a pharmacological potential for the treatment of kidney diseases is being widely investigated in both clinical and non-clinical studies; thus, the control of factors that inhibit this antioxidant response in CKD, such as IS, are of primary importance in controlling inflammation and oxidative stress in these patients [3,53,54]. 

Macrophage function, as for other immune cells, was shown to be impaired by uremic toxins [14,15,16,17,18]. In peritoneal macrophage derived from IS-treated mice we also observed a pro-inflammatory state due to COX-2 and iNOS expression and an increased nitrotyrosine formation. Moreover, similarly to the results obtained in the intestinal tissue, IS also induced a significant increase of the proapoptotic protein Bax in these cells, thus further contributing to an impaired macrophage function. These data are in according with previous data concerning the pro-oxidant and pro-inflammatory effect of IS in other systems [27,55,56], thus supporting the effect of IS on many organs, and then its role in many CKD-associated complications. In fact, this effect observed in macrophages could have a particular relevance both in the altered immune response and in the cardiovascular complication observed in CKD considering that persistent inflammation is on one hand, per se, a risk factor for CKD progression but on the other may also modulate the impact of other vascular and nutritional risk factors in the toxic uremic milieu [4].

Several uremic metabolites and their precursors exert immunomodulatory effects both at their side of origin and especially in the circulation [11,12]. The upregulation and presence of cytokines such as TNF-α, IL-1β and IL-6, in the blood contribute to chronic inflammation and resulted increased in CKD patients. [57,58]. 

Serum mice analysis indicated a significant increase in TNF-α, IL-6 and IL-1β in IS-treated animals thus indicating that IS significantly contributes to the systemic increase of these cytokines and to a condition of systemic inflammation. Interestingly, this effect is more pronounced after 6 h of treatment supporting the IS proinflammatory effect when the serum concentrations decrease. Although cytokine production is necessary for protection against pathogens and promote tissue repair, excessive release or decreased clearance, or both, can lead to organ failure and premature death. Indeed, these cytokines are listed in databases of uremic toxins and uremic retention solutes [59], thus, understanding how the pro-inflammatory cytokines are induced and reducing their synthesis may represent a future strategy for therapeutic prevention of many CKD complications, such as recurrent infection, sepsis, and cardiovascular disease in patients with chronic renal failure [60].

Our results indicated that among the various uremic toxins accumulating in CKD patients, IS significantly contributes to intestinal and systemic inflammation observed in CKD, thus, highlighting IS as a potential pharmacological target in this disease.

## 4. Materials and Methods

### 4.1. In Vivo Studies

#### 4.1.1. Reagents 

Unless stated otherwise, all reagents and compounds were purchased from Sigma Chemicals Company (Sigma, Milan, Italy). 

#### 4.1.2. Animals 

Female C57BL/6J mice (6–8 weeks; 20–25 g; Charles River Laboratories, Lecco, Italy) were housed in a controlled environment facility at the University of Salerno, Department of Pharmacy. The mice were housed with a 12:12-h light/dark cycle and received a standard chow diet and water ad libitum.

The animal experiments were performed in compliance with Italian regulations on protection of animals used for experimental and other specific purposes (D.M.116192) as well as with European Economic Community regulations (O.I. of E.C. L 358/1 12/18/1986). 

The University of Salerno Review Board for Animal Care (OPBA) and the Ministry of Health approved the study (n. 488/2018-PR). IS was dissolved into a saline solution (vehicle) and it was intraperitoneally injected into mice (800 mg/kg, i.p. given once) [24,34]. After 3 h or 6 h of treatment, animals were sacrificed, and the ileum, colon peritoneal macrophages, and serum were collected and stored for the analysis.

#### 4.1.3. Experimental Groups

C57BL/6J mice were randomly divided into the following groups (*n* = 12):

- SHAM + vehicle group: vehicle solution was given by i.p.
- IS 3 h (800 mg/kg; i.p.) group: IS was administered i.p. for 3 h
- IS 6 h (800 mg/kg; i.p.) group: IS was administered i.p. for 6 h;

#### 4.1.4. IS Serum Evaluation by HPLC

The IS levels in mice serum were evaluated according the methods of Zhu et al. [61] as previously reported [24].

#### 4.1.5. Histopathological Analysis

Intestines were collected during necropsy of 19 C57BL/6J mice. The samples were fixed in 10% neutral buffered formalin and embedded in paraffin. Transversal sections at 4 μm were stained with hematoxylin and eosin. 

Sections were evaluated and histological findings were scored by two independent pathologists (F.P. and O.P.) under an optical microscope (Nikon E600; Nikon, Tokyo, Japan) in a blinded fashion. Discordant results were reviewed with a multiheaded microscope to reach consensus.

The severity of intestinal inflammation has been scored with an established method, according to the leukocyte density of lamina propria area infiltrated: absent (score 0) 0% of the lamina propria area is infiltrated; mild (score 1) <10%; moderate (score 2) 10–25%; severe (score 3) >26% [62].

Villi were considered blunted when villous-to-crypt-length ratio was less than or equal to 2:1 [62]. Villi were considered fused when a partial or complete fusion of contiguous villi was evident. Blunted and fused villi were scored as follow: absent (score 0) 0% of villi were blunted or fused; mild (score 1) <10%; moderate (score 2) 10–25%; severe (score 3) >26%.

The infiltration of inflammatory cells in the mesentery (peritonitis) has been scored as follow: absent (score 0) no inflammatory cells observed; mild (score 1) rare scattered (<5) inflammatory cells per 40× HPF; moderate (score 2) 5–10 inflammatory cells per 40× HPF; severe (score 3) more than 10 inflammatory cells per 40× HPF.

#### 4.1.6. Immunohistochemistry 

Sections at 4 μm were deparaffined in xylene and reiterated in the decreasing series of alcohol. Peroxidases were blocked with a solution of hydrogen peroxide and methanol (4:1) for 15 min. Antigen retrieval pretreatments were performed using a HIER citrate buffer pH 6.0 (Bio-Optica, Milan, Italy) for 20 min at 98 °C. Subsequently, the antibodies with rabbit host and mouse host followed two different protocols. Shortly, immunohistochemistry was performed according to the protocols described by De Biase et al. [63]. For antibodies with a rabbit host, immunohistochemistry was performed according to the protocol indicated by the MACH1 Universal HRP-Polymer Detection Kit (Cat. No: M1U539 G, L10, Bio-Optica, Milan, Italy). As primary antibodies, we used Polyclonal Rabbit Anti-COX-2 (Cat. No: 12375-1-AP, Proteintech, Rosemont, IL, USA) at 1:100 in PBS and Polyclonal Rabbit Anti-Nitrotyrosine (Code n. 06-284, Merck, Darmstadt, Germany) at 1:500 in PBS. For antibodies with mouse host, immunohistochemistry was performed according to the protocol indicated by the M.O.M. Kit (Cat. No: PK-2200, Vector Laboratories, Burlingame, CA, USA) [64]. As the primary antibody, we used a mouse monoclonal antibody Anti-Bax (Clone 6A7, Santa Cruz Biotechnology, Dallas, TX, USA) at 1:50 in PBS.

DAB stain was quantified with FIJI (ImageJ, National Institutes of Health). For each case, 3 random 40× fields of the transverse section of the intestine were photographed under an optical microscope (Nikon E600; Nikon, Tokyo, Japan) associated with a digital camera (Nikon digital camera DMX1200).

For the markers expressed both by IEC-6 cells and by infiltrating inflammatory cells (Nitrotyrosine and Bax) the positive tissue area was measured. The function “Color threshold” was used on each row image to measure the total area of the analyzed tissue (Hue 0–255, Saturation 0–255, Brightness 0–230). To evaluate the positive area, each raw image was processed with the Color Deconvolution function using the preset Hematoxylin + DAB settings. A threshold (0–70) was applied to the brown channel images to remove background signal. Finally, the positive area was measured [65]. The percentage of positive tissue was calculated for each case and expressed as relative to the higher percentage of positive tissue measured.

COX-2 was expressed only by infiltrating inflammatory cells and its expression was evaluated by counting the number of positive cells. After processing row images with Color Deconvolution and threshold as above described, positive cells were counted with the Automatic Particle counting function with the following settings: range of area between 100 and 400 µm^2^ and a range of circularity between 0.3 and 1 [66]. The total number of positive cells for each case was expressed as relative to the higher number of cells measured in a single case.

### 4.2. In Vitro Studies 

#### 4.2.1. Cell Culture

The IEC-6 cell line (CRL-1592) was purchased from the American Type Culture Collection (ATCC, Rockville, MD, USA), and derived by normal rat small intestinal epithelial crypt. IEC-6 cells are routinely maintained in the presence of Dulbecco’s modified Eagle’s medium (DMEM; 4 g/L glucose) containing 10% (*v*/*v*) fetal bovine serum (FBS), 2 mM L-glutamine, 1.5 g/L NaHCO_3_, and 0.1 U/mL bovine insulin. Thecells were grown at 37 °C in a humidified atmosphere of 5%CO_2_/95% air and viability was monitored using phase contrast microscopy and trypan blue staining. For the experiments, IEC-6 cells were used between the 16th and 19th passages [67].

#### 4.2.2. Cellular Treatment

In order to estimate the pro-inflammatory activity of IS, the IEC-6 cells were plated and, after 24 h of adhesion, were treated with IS (125–1000 µM) alone or in presence of pro-inflammatory stimuli, such as lipopolysaccharides from *E. coli* (LPS; serotype O111:B4; 10 µg/mL) plus interferon-γ (IFN; 10 U/mL), for different times, depending on the mediator to evaluate.

#### 4.2.3. TNF-α Determination

TNF-α levels were measured in IEC-6 cells medium (8.0 × 10^4^ cells/well/24-well plates), treated with IS (125–1000 µM) alone and in presence of LPS + IFN, by an Enzyme-Linked Immuno Sorbent Assay (ELISA) according to the manufacturer’s instructions (e-Biosciences, San Diego, CA, USA). The results were expressed as pg/mL, as formerly reported [68].

#### 4.2.4. Evaluation of COX-2 and iNOS Expression and Nitrotyrosine Formation by Flow Cytometry Analysis

In order to evaluate the involvement of the IS in the exacerbation of the inflammatory state, IEC-6 cells were plated in 96-wells plates (2.0 × 10^3^ cells/well) and, after adhesion, were treated with IS (125–1000 µM) alone and in presence of pro-inflammatory stimuli, such as LPS + IFN, for 24 h. The cells were then collected and washed twice with phosphate buffered saline (PBS) then a fixing solution was added to the IEC-6 cells for 20 min and after the cells were incubated in fix perm solution for a further 30 min. Anti-cyclooxygenase-2 (COX-2; BD Transduction Laboratories, Milan, Italy), anti-inducible nitric oxide synthase (iNOS; BD Transduction Laboratories, Milan, Italy), or anti-nitrotyrosine (Merck Millipore, Milan, Italy) antibodies were then added for 1 h. The secondary FITC-conjugate antibody was added to IEC-6 cells in fixing solution and cell fluorescence was evaluated by a fluorescence-activated cell sorter (FACSscan; Becton Dickinson, Milan, Italy) and then elaborated by Cell Quest software (version 4; Becton Dickinson, Milan, Italy), as previously reported [69]. 

### 4.3. Ex Vivo Studies

#### 4.3.1. Primary Murine Peritoneal Macrophages

Peritoneal cells were harvested by means of lavage of the peritoneum with 5 mL of EDTA 0.5 mM plated and allowed to adhere for 2 h at 37 °C in a 5% CO_2_ atmosphere [27]. Subsequently, non-adherent cells were removed and RPMI 1640 medium with 10% FBS was added. The cells were maintained in culture for 24 h at 37 °C in a 5% CO_2_ atmosphere before experiments.

#### 4.3.2. Measurement of Intracellular Reactive Oxygen Species (ROS) Release

Reactive oxygen species production was evaluated by means of the probe H_2_DCF-DA as previously reported [70]. H_2_DCF, in presence of ROS, is rapidly oxidized to the highly fluorescent DCF. Following treatment with IS for 3 and 6 h, primary murine peritoneal macrophages (3.0 × 10^5^ cells/well) were collected, washed with PBS and incubated in PBS containing H_2_DCF-DA (10 µM) at 37 °C, for 15 min. Cellular fluorescence was evaluated using fluorescence-activated cell sorting analysis (FACSscan; Becton Dickinson) and elaborated with Cell Quest software.

#### 4.3.3. Detection of HO-1, SOD-2, COX-2, iNOS and Bax Expression and Nitrotyrosine Formation by Cytofluorimetry

Cytofluorimetric analysis was performed as previously reported [71]. Briefly, after the IS-treatment for 3 and 6 h, and after further 24 h in presence of LPS (1 µg/mL), primary murine peritoneal macrophages (5.0 × 10^4^ cells/well) were collected, washed with PBS, incubated in fixing solution for 20 min, and then in fix perm solution for 30 min at 4 °C. Anti-heme oxygenase-1 antibody (HO-1; Santa Cruz Biotechnologies), anti-superoxide dismutase antibody 2 (SOD-2; Santa Cruz Biotechnologies), anti-COX-2 antibody (BD Transducion Laboratories), anti-iNOS antibody (BD Transducion Laboratories), anti-nitrotyrosine antibody (Merck Millipore, Milan, Italy) or anti-Bax antibody (Santa Cruz Biotechnologies), were added to primary murine peritoneal macrophages. The secondary antibody (Immuno Reagents) was added in Fix Perm Solution and the cells were evaluated using a fluorescence-activated cell sorting (FACSscan; Becton Dickinson) and elaborated with CellQuest software. 

#### 4.3.4. TNF-α, IL-1 and IL-1β Serum Levels

Cytokines levels were evaluated on serum samples of mice treated with IS (800 mg/kg) for 3 or 6 h. For this analysis, we used commercially available kits for murine TNF-α, IL-6 and IL-1β (ThermoFisher Scientific, Carlsbad, CA, USA) as previously reported [24]. 

### 4.4. Data Analysis and Statistical Evaluation

Data are given as the standard error of the mean (s.e.m.) showing the combined data of at least three independent experiments each in triplicate. Statistical analysis was performed by an analysis of variance test, and multiple comparisons were made by Bonferroni’s test. A *p*-value lower than 0.05 was considered significant. 

For histopathological and immunohistochemical experiments, the analysis of variance was made with one-way ANOVA test and differences between studied groups were evaluated with Tukey’s test using Prism 7.0 (GraphPad Software, San Diego, CA, USA).

## 5. Conclusions

Our study indicated that IS induces intestinal inflammation and oxidative stress, also affecting IECs and activating peritoneal macrophages, in terms of inflammation, oxidative stress, and apoptosis. These effects are also associated with an increase of pro-inflammatory cytokines, in mice serum, providing evidence for the systemic effects of IS in CKD patients. These observations support the role of IS in CKD-associated complications, and the importance of its control (e.g., with diet or oral adsorbent) in these patients. 

## Data Availability

The data presented in this study are available on request from the corresponding author.
